# Supplementary material for: The effect of big data technologies usage on social competence
Source: PeerJ Comput Sci. 2023 Nov 17;9:e1691. doi: 10.7717/peerj-cs.1691 (PMC10703065; doi:10.7717/peerj-cs.1691)
Supplement: Supplemental Information 2 [file peerj-cs-09-1691-s002.docx]

**Electronic Social Competence Scale**

| *Card domains* | | *Strongly disagree* | *Disagree* | *Neither agree nor disagree* | *Agree* | *Strongly agree* |
| --- | --- | --- | --- | --- | --- | --- |
| Operational Definition | | | | | | |
| 1 | I can face challenges in the e-learning environment by thinking about them in a way that helps me stay calm. |  |  |  |  |  |
| 2 | I can increase my positive feelings when engaging in a chat room interaction by changing the way I think about the situation. |  |  |  |  |  |
| 3 | I control my emotions by changing the way, I think in a situation that requires dealing with educational videos. |  |  |  |  |  |
| 4 | When I want to reduce my negative feelings while taking online tests, I change the way I think about the question. |  |  |  |  |  |
| 5 | My colleagues and friends reach out to me through social media when they have a problem. |  |  |  |  |  |
| 6 | My colleagues and friends help me through private messages when I need them. |  |  |  |  |  |
| 7 | My colleagues interact with me in educational forums. |  |  |  |  |  |
| 8 | My colleagues feel comfortable working with me electronically. |  |  |  |  |  |
| 9 | My colleagues and friends know that they can count on me when they have to organize some kind of online activity. |  |  |  |  |  |
| 10 | I participate in online activities that others participate in. |  |  |  |  |  |
| 11 | I feel that there is a similarity between me, and my colleagues when using the learning management system. |  |  |  |  |  |
| 12 | I feel like I have friends in electronic environments. |  |  |  |  |  |
| 13 | If one of my colleagues in the chat room is confused and does not have time to finish his work, I give him a hand. |  |  |  |  |  |
| 14 | I am standing up for my mates who are being mocked in the chat room. |  |  |  |  |  |
| 15 | When a colleague or friend is sad, I comfort them with private messages to make them feel better. |  |  |  |  |  |
| 16 | When I see a fellow chat room feeling left out and alone, I help them blend in with my group of friends. |  |  |  |  |  |
| 17 | I help my classmates who have physical problems to perform electronic activities. |  |  |  |  |  |
| 18 | In relationships with my colleagues on the forums, I feel very active. |  |  |  |  |  |
| 19 | In relationships with my teachers in chat rooms, I feel effective. |  |  |  |  |  |
| 20 | In relationships with my family while studying remotely, I feel I am doing things well. |  |  |  |  |  |
| 21 | Distance learning makes my relationship with the elders in my family more effective. |  |  |  |  |  |
| 22 | I let my colleagues finish their electronic interventions without disturbing them. |  |  |  |  |  |
| 23 | I ask permission to speak and wait for my turn to speak. |  |  |  |  |  |
| 24 | I follow the rules set by my teachers to participate in the online environment. |  |  |  |  |  |
| 25 | I respect other people's opinions even if they do not coincide with mine. |  |  |  |  |  |
| 26 | I treat learning management system tools, and educational videos with respect. |  |  |  |  |  |
